# Vapor-Assisted Crosslinking of a FK/PVA/PEO Nanofiber Membrane

**DOI:** 10.3390/polym10070747

**Published:** 2018-07-06

**Authors:** Jiao Ding, Man Chen, Wenjie Chen, Ming He, Xiangyang Zhou, Guoqiang Yin

**Affiliations:** 1 College of Chemistry and Chemical Engineering, Zhongkai University of Agriculture and Engineering, Guangzhou 510225, China; 13726706692@163.com (M.C.); 18825070459@163.com (W.C.); heming1026@163.com (M.H.); zhouxyzps@163.com (X.Z.); 2 Guangzhou Key Laboratory for Efficient Utilization of Agricultural Chemicals, Guangzhou 510225, China

**Keywords:** keratin, nanofiber membrane, electrospinning, vapor-assisted, crosslinking

## Abstract

Herein, we demonstrate a three-component FK/PVA/PEO nanofiber membrane by electrospinning and vapor-assisted crosslinking. We have used feather-derived-keratin (FK), poly (vinyl alcohol) (PVA), and poly (ethylene oxide) (PEO) as membrane components and citric acid/glyoxal as the crosslinker. The structural, thermal, hydrophobicity, and mechanical properties of the as-prepared and crosslinked FK/PVA/PEO nanofiber membranes have been systematically investigated. The results suggest that the nanofiber membrane’s vapor-assisted crosslinking by citric acid has shown better performances than that of glyoxal used as a crosslinker. These results exhibit that non-toxic citric acid can be used as a crosslinking agent to modify the performance of keratin-based membranes. This study opens up further avenues for post-synthesis modification of polymeric membranes for a wide range of applications.

## 1. Introduction

Keratin is a kind of structural fibrous protein, which is derived from the ectoderm cells of the animal and offers excellent biocompatibility, degradability and skin-friendly properties [1]. Moreover, keratin does not cause skin irritation and promotes wound tissue healing, making it an ideal candidate for biomedical applications, such as biomaterials and wound dressings [2]. Usually, keratin is extracted from wool, human hair and chicken feathers [2]. The keratin content in chicken feathers can reach up to 90%, but most of the chicken feathers are trashed. However, a very small fraction of the feathers is used in agriculture, textiles, the beauty industry, and as biomedical materials. Therefore, the utilization of chicken feathers for keratin production is an eco-friendly and cost-effective approach. Moreover, feather-derived-keratin (FK) can reduce the environmental pollution and protein resource waste [3]. The recovery of valuable chemicals from waste is a highly sought-after sustainable approach. Didaskalou et al. [4] present the development and modelling of a continuous adsorption process with in situ solvent recovery for the isolation of oleuropein from olive leaves, an agricultural waste. Cucina et al. [5] investigated the feasibility of energy and plant nutrients recovery from the pharmaceutical organic waste originated from a fermentative biomass used for the daptomycin production.

Electrospinning is an established technique for the fabrication of micro and nanofibers [6]. The electrospinning nanofiber membrane offers large specific surface area, high porosity, uniform pore size, and excellent gas exchange properties [7]. The structure of the nanofiber membrane, prepared by combining natural protein with electrospinning, is similar to the natural extracellular matrix, which is the best material to mimic the extracellular matrix environment.

The hydrophilic polymers, poly (vinyl alcohol) (PVA) and poly (ethylene oxide) (PEO), offer an excellent array of properties, such as non-toxicity, biodegradability, biocompatibility, and mechanical performance. In addition, the formation of the nanofiber membrane from these polymers is facilitated by the electrospinning process due to the high molecular weight and large viscosity of PVA and PEO. Recently, the research has focused on the development of protein nanofiber membranes by using these hydrophilic polymers and keratin materials [8,9]. However, the structure of the nanofiber membrane prepared by blending keratin and hydrophilic polymers, such as PVA and PEO, is destroyed due to water absorption and swelling. In addition, the thermal performance and mechanical properties of blended nanofibers are poor. Hence, the choice of crosslinker plays a critical role in crosslinking modifications.

Crosslinked polymer membranes have potential applications in nanofiltration. Therefore, crosslinking is often used to prepare solvent-resistant membranes for nanofiltration. Valtcheva et al. [10] prepared polybenzimidazole (PBI) membranes and crosslinked with dibromoxylene (DBX) for organic solvent nanofiltration (OSN). Fei et al. [11] developed mixed matrix membranes comprising of graphene oxide and polybenzimidazoles for nanofiltration in organic media.

Commonly, a large number of aldehyde crosslinkers, such as formaldehyde, glyoxal, and glutaraldehyde, are used in the chemical crosslinking modification of protein materials. Keratin is rich in amino and carboxyl groups, and reacts with aldehyde crosslinkers to form crosslinking bridges, which results in extensive networking. Park et al. [12] have studied the effect of different glyoxal content on the properties of keratin/PVA nanofibers and showed that water resistance, thermal stability, and mechanical properties of the composite membranes had been greatly improved after glyoxal crosslinking. Despite the positive influence of aldehyde crosslinkers on composite membrane performance, the toxic nature and difficulty in direct electrospinning limit their utilization. The aldehyde crosslinkers cannot be directly added to the electrospinning solution due to their high reaction activity. Additionally, the aldehyde crosslinkers are toxic, and the residual in nanofiber membrane may cause safety hazard [13]. Few reports have adopted citric acid as a crosslinking agent due to its safety, reliability, and cost-effectiveness [14]. For instance, Wang et al. [15] have fabricated keratin/carboxymethylcellulose sodium blend membranes by using citric acid as a crosslinking agent. Wang et al. [15] have demonstrated that the water resistance and mechanical properties of the blend membranes have been significantly improved by adding an optimal amount of citric acid.

However, most of these studies have used a two-component system for electrospinning and, to the best of our knowledge, a three-component system has not been studied for nanofiber membrane fabrication. Herein, we have demonstrated the fabrication of FK/PVA/PEO three-component nanofiber membranes by the electrospinning process. Moreover, the vapors of citric acid and glyoxal were utilized to crosslink the as-prepared nanofiber membranes, instead of directly adding them into the electrospinning solution. The morphology, structure, thermal stability, water resistance, and mechanical properties of the as-prepared and vapor-assisted crosslinking membranes were evaluated to study the influence of crosslinkers. The fabricated FK/PVA/PEO nanofiber membranes and vapor-assisted crosslinking modification are expected to broaden the utilization of protein membranes in the field of polymeric materials.

## 2. Materials and Methods

### 2.1. FK Extraction

The chicken feathers were collected from the waste market. The FK was extracted by following the same process, as described elsewhere [16]. After washed and unbleached, the chicken feathers were added to 16% freshly prepared peracetic acid solution (1:12, *w*/*w*) and the extraction was performed at 60 °C for 100 min under constant stirring. Then the extraction FK solution was dialyzed using dialysis bags (Oso-T8280, 12,000–14,000 Da; Union Carbide, Houston, TX, USA) in distilled water at room temperature for a week, and the water was changed every 24 h. FK was precipitated through dialysis. After filtration, washing and drying, FK was ground and preserved in a sealed bag before usage. The as-prepared FK powder had a faint-yellow color and the molecular weight of the as-prepared FK is above 14,000 Da.

### 2.2. Electrospinning of the FK/PVA/PEO Nanofiber Membrane

The electrospinning solution consists of FK, PVA, and PEO solutions. The FK solution (12 wt %) was prepared by adding as-prepared FK powder into the distilled water under continuous stirring at 50 °C. The pH value of the FK solution was adjusted to 8 by adding 3 mol·L^−1^ NaOH. The 12 wt % PVA and 12 wt % PEO solutions were prepared by dispersing the required amounts of corresponding powders in distilled water under continuous stirring at 80 °C. To obtain the electrospinning solution, 3 g of FK solution was mixed with 8.4 g of PVA solution and 3.6 g of PEO solution. The electrospinning solution was thoroughly mixed under vigorous stirring and undesirable bubbles were removed by ultrasonication.

The FK/PVA/PEO three-component nanofiber membrane was prepared by using an electrospinning machine (ET-2535DC, Beijing Yongkang Leye Technology Development Co. Ltd., Beijing, China) with a constant flow rate of 0.08 mL/h, an accelerating voltage of 18 kV and the distance of 10 cm from the syringe tip to collector, which was covered with aluminum foil.

### 2.3. Vapor-Assisted Crosslinking of the FK/PVA/PEO Nanofiber Membrane

The FK/PVA/PEO nanofiber membrane was crosslinked by using vapors of citric acid and glyoxal, respectively. A total of 200 mL of saturated citric acid solution and 200 mL of glyoxal solution (40 wt %) were placed in two closed containers, covered with a metallic mesh. The FK/PVA/PEO membranes were placed on top of the metallic mesh. The vessels, containing citric acid and glyoxal, were heated and maintained at 60 °C. The vapors of citric acid and glyoxal were used to crosslink the FK/PVA/PEO nanofiber membrane. After vapor-assisted crosslinking, the FK/PVA/PEO nanofiber membranes were placed in the fume hood under air, for 2 h, to remove the residual crosslinker. The membranes were stored in a dry, sealed bag. The FK/PVA/PEO nanofiber membranes were treated with citric acid vapors and glyoxal vapors for 3 h and 12 h and referred to as 3h-C, 12h-C, 3h-G, and 12h-G, respectively.

### 2.4. Characterization

The surface morphology of the nanofiber membranes was observed by scanning electron microscopy (SEM, EVO 18; Carl Zeiss, Oberkochen, Germany). The average diameter of the nanofibers was measured by image analysis software (Image-Pro Plus, Media Cybernetics, Maryland, MD, USA) and 100 nanofibers were randomly selected from each sample. The structure of the nanofiber membrane was confirmed by FTIR (spectrum 100; Perkin-Elmer, Waltham, MA, USA), carried out in a range of 4000–550 cm^−1^. The X-ray diffraction analysis was carried out by an X-ray diffractometer (XRD, Almelo, Empyrean, Holland) with Cu Kα (λ = 1.540 Å) radiations The XRD spectra were recorded in the 2θ range of 5° to 50° at a step of 0.02°. The thermal stability was assessed by thermogravimetric analysis (TGA, Instruments TG209F1, Netzsch, Bavaria, Germany) in the temperature range of 40 to 700 °C with a heating rate of 10 °C/min. The temperature was increased at the heating rate of 10 °C/min. Differential scanning calorimetry (DSC) was also applied to analyze the thermal property of the nanofiber membranes by using a differential scanning calorimeter (Mettler Toledo, Zurich, Switzerland). The water contact angle (WCA) of the nanofiber membrane was obtained by using the sessile drop method at room temperature. The contact angle measurements were carried out by using a DSA100 instrument (KRUSS, Hamburg, Germany). The mechanical properties of the nanofiber membranes, such as tensile strength (σ_b_) and elongation at fracture (ε_b_), were determined by using a universal testing machine (CMT6503, Shenzhen MTS Test Machine Company Ltd., Shenzhen, China). The tensile measurements were carried out by using the same protocol as described elsewhere [17].

## 3. Results

### 3.1. Microstructural Analysis

**Figure 1:**
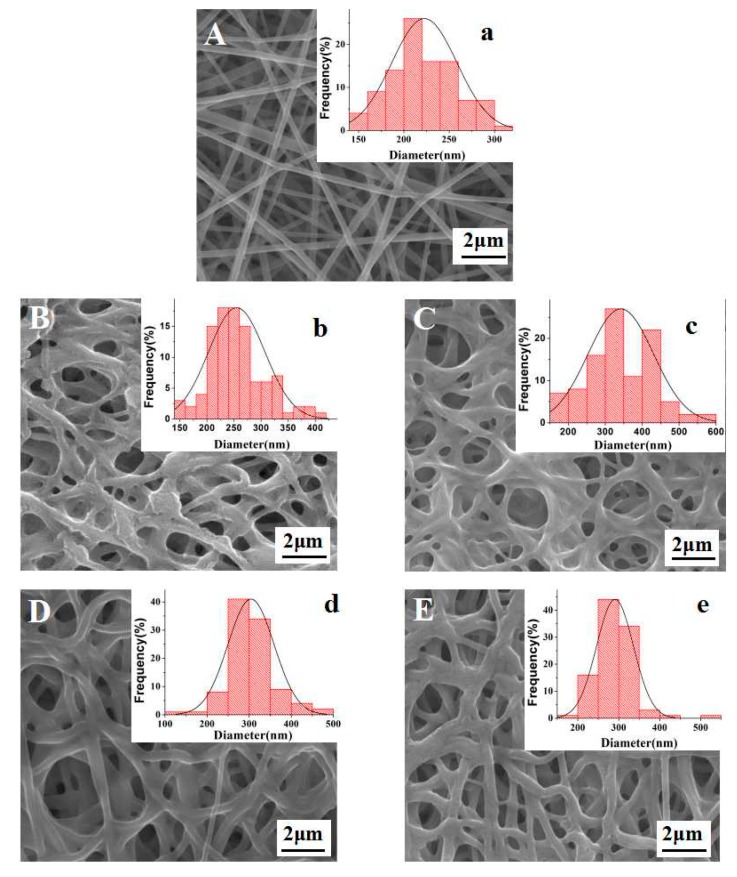
SEM images and diameter distributions of FK/PVA/PEO nanofiber membranes: (**A**) without treatment; (**B**) 3h-C; (**C**) 12h-C; (**D**) 3h-G; and (**E**) 12h-G. The insets show the diameter-distribution of corresponding samples (for example, 3h-C refers to the FK/PVA/PEO nanofiber membranes crosslinked by citric acid vapor for 3 h. C and G are the English initials abbreviation of citric acid and glyoxal, respectively; 3 h is the crosslinking time).

Figure 1 presents the SEM images and diameter-size distribution of the as-prepared and crosslinked membranes. It can be seen from Figure 1A(a) that the as-prepared FK/PVA/PEO nanofiber membrane exhibited randomly oriented, bead-free, smooth and uniform nanofibers. The average diameter of nanofibers was 223 ± 36 nm (Table 1). Moreover, we have not observed any obvious phase segregation in as-prepared FK/PVA/PEO three-component nanofiber membrane, which implies the excellent compatibility of these three components. After vapor-assisted crosslinking, the FK/PVA/PEO nanofiber membranes have maintained the fibrous structure, however, the distance between nanofibers has been significantly decreased (Figure 1). Furthermore, the average diameter of nanofibers after vapor-assisted crosslinking increased with reacting time. The SEM images of 3h-C and 12h-C depicts that the average diameter has increased, whereas the distance between nanofibers has decreased by increasing the reaction time, which can be attributed to the presence of carboxyl and hydroxyl groups in citric acid. The reactions of carboxyl and hydroxyl groups with amino and carboxyl groups from FK led to the growth of molecular chains, which resulted in the increased nanofibers diameter [15]. The SEM images of 3h-G and 12h-G which crosslinked by glyoxal vapor show that an increase in diameter and decrease in distance between nanofibers with reaction time (Table 2), but the trend of the change is lower than that of treated with citric acid vapor. It is worth mentioning that the small molecules of glyoxal can diffuse into the FK/PVA/PEO nanofibers and react with FK and PVA. As a result, the structure of the crosslinking network was formed between macromolecules, which led to a more compact buildup [18]. 

### 3.2. Structural Analysis

**Figure 2:**
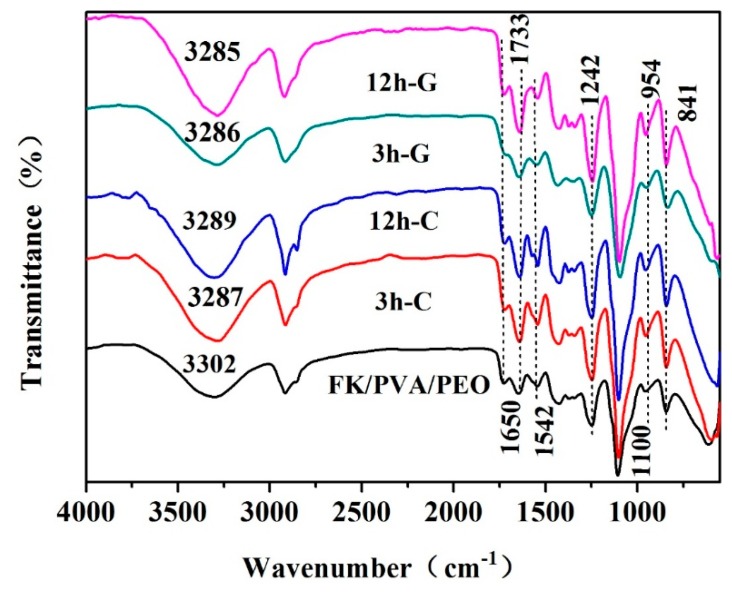
The FTIR spectra of as-prepared and crosslinked FK/PVA/PEO nanofiber membranes.

Figure 2 shows the FTIR spectra of the as-prepared and crosslinked FK/PVA/PEO nanofiber membranes. A typical adsorption band of peptide bonds (–CONH–) was observed in Figure 2, which is a clear signature of FK presence. The vibrations of the peptide bonds can be attributed to amide A, amide I, amide II and amide III [19,20]. The broad absorption band at ~3300 cm^−1^ corresponds to amide A. The adsorption peak at 3302 cm^−1^ represents the stretching vibrations of N–H bond. The amide I bands, due to C=O stretching vibration, were observed at 1650 cm^−1^. The amide II can be represented by a sharp peak at 1542 cm^−1^, which is related to the N–H bending vibrations. Moreover, a sharp peak at 1242 cm^−1^ corresponds to the characteristic absorption peak of the amide III (1220–1300 cm^−1^). The peak at 1427 cm^−1^ refers to the O–H and C–H bending vibrations of PVA [21]. The absorption bands, corresponding to CH_2_ and C–O–C of PEO, can be evidenced by sharp peaks at 2923 and 1100 cm^−1^, respectively [22]. In addition, the absorption peaks at 954 and 841 cm^−1^ represent the planar structure of PEO [20]. These peaks correspond well to the established FTIR spectra of FK, PVA and PEO, which confirms that we have successfully synthesized FK/PVA/PEO membranes.

After crosslinking, the absorption peak at ~3300 cm^−1^ has been slightly shifted to a lower wavenumber, which suggests that crosslinking can effectively enhance the intermolecular interaction of composite membranes [23]. We have not observed any additional peak after vapor-assisted crosslinking treatment. One should note that the amide I band, due to the C=O stretching vibration of FK, coincides with the vibration peak at 1730 cm^−1^ of ester (–OCO–), which is formed by the reaction of citric acid vapors and FK. Moreover, the amide I band of FK also covered the stretching vibration peak at 1640 cm^−1^ (C=N) of the Schiff base, formed by glyoxal vapors and FK.

In summary, the FTIR spectra have shown the characteristic absorption bands of FK, PVA, and PEO from the composite nanofiber membrane. The FTIR analysis demonstrates that the vapor-assisted crosslinking of FK/PVA/PEO membrane does not destroy the structure of any membrane component due to their excellent compatibility.

**Figure 3:**
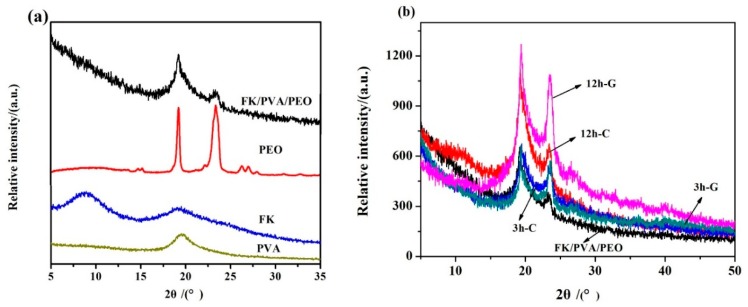
The XRD spectrums of (**a**) individual components (FK, PVA and PEO); and (**b**) the as-prepared membrane and crosslinked membranes.

The XRD spectra of the pure components (FK, PVA, and PEO), as-prepared FK/PVA/PEO nanofiber membrane, and vapor-assisted crosslinked FK/PVA/PEO nanofiber membranes are shown in Figure 3. The broad diffraction peaks at 2θ = 9° and 19° correspond to the α-helix and β-sheet structure of FK, respectively [21]. Moreover, the broad nature of XRD peaks indicates the partially crystalline nature of the prepared material. The XRD pattern of PVA gives a typical diffraction peak at 2θ = 19° [19]. The sharp diffraction peaks at 2θ = 19° and 23° indicate the highly crystalline nature of PEO [24]. The XRD pattern of FK/PVA/PEO nanofiber membrane has exhibited the similar pattern as of FK, PVA, and PEO. The sharp diffraction peaks were observed at 2θ = 19° and 23°. The broad peak at 2θ = 9°, corresponding to FK, did not appear in the XRD pattern of FK/PVA/PEO membranes. Moreover, the lower intensity of the peak at 2θ = 23° suggests that the overall crystallinity of the FK/PVA/PEO nanofiber membrane is slightly lower than PEO. The results show that pure components are not just physically mixed, but strong molecular interactions have been generated. Hence, the regularity and crystalline structure of the original molecules has been reduced.

The XRD patterns of crosslinked membranes indicate that crosslinking time has a direct relationship with the crystallinity of the FK/PVA/PEO nanofiber membrane (Figure 3b) [25]. Furthermore, we have not observed the formation of impurity phases after the vapor-assisted crosslinking process. The results suggest that two kinds of crosslinkers reacted with the FK/PVA/PEO nanofiber membrane rather than simply mixed with the three components.

### 3.3. Thermal Analysis

**Figure 4:**
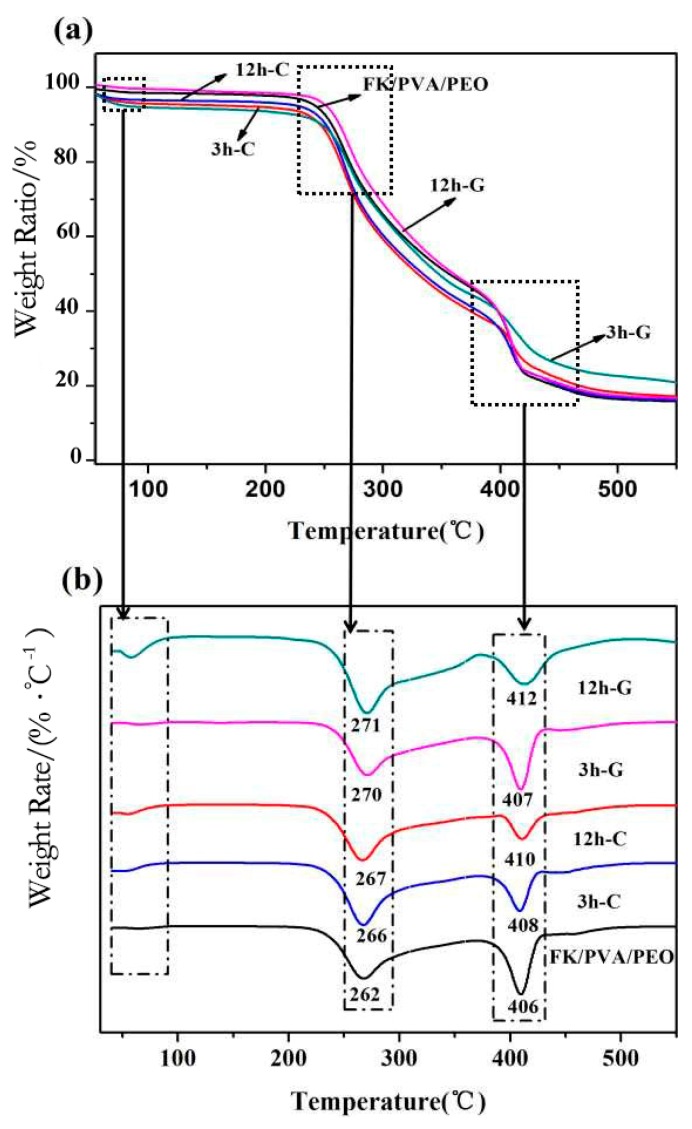
The thermal analysis of as-prepared and crosslinked FK/PVA/PEO nanofiber membranes: (**a**) TGA curves; and (**b**) DTG curves.

As can be seen from Figure 4a,b, the weight loss process of the nanofiber membrane can be divided into three stages. The decomposition temperature of the first stage is below 100 °C, which is caused by evaporation of water.

The weight loss of 5% was observed due to excellent hydrophilicity of PVA and PEO. In the second stage, the weight loss of 53% was observed below the decomposition temperature is about 260 °C, which can be attributed to the thermal decomposition of FK and PVA in nanofiber membrane. In the third stage, the weight loss of 83% occurred below 400 °C, which corresponds to the thermal decomposition of PEO and the blended nanofiber membrane. The thermal decomposition behavior of the nanofiber membrane is shown more clearly by using DTG curves (Figure 4b). We have observed that the decomposition temperature of the crosslinked FK/PVA/PEO nanofiber membrane is higher than the as-prepared FK/PVA/PEO nanofiber membrane. In addition, the decomposition temperature slightly increased with the increase in the reaction time. Furthermore, the area of the decomposition peaks for the crosslinked membrane has been reduced, which can be attributed to the intramolecular network structure by crosslinking modification. However, in the third stage, the area of the decomposition peak for citric-treated membranes is smaller than glyoxal-treated membranes, which implies that citric acid treatment offers the higher thermal stability of FK/PVA/PEO nanofiber membranes. The results demonstrate that the vapor-assisted crosslinking of FK/PVA/PEO nanofiber membranes can effectively enhance the thermal stability of the blend membrane.

**Figure 5:**
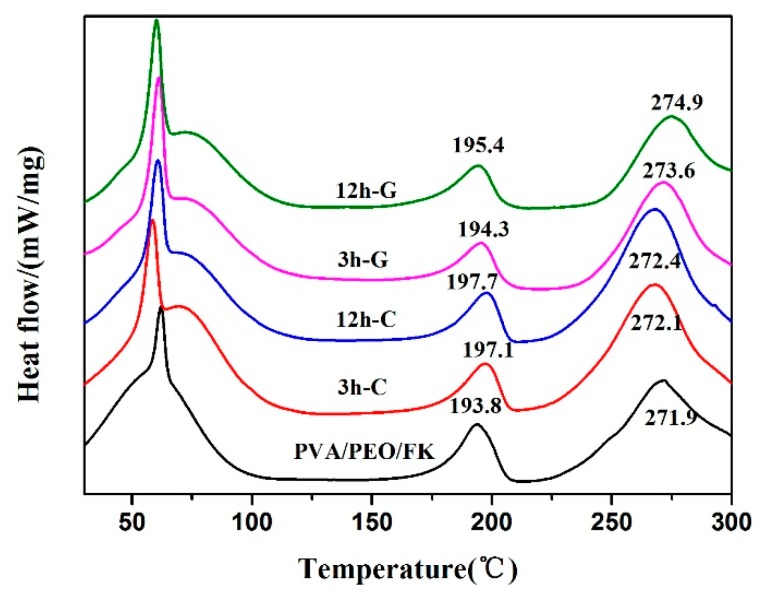
The DSC curves of the as-prepared and crosslinked FK/PVA/PEO nanofiber membranes.

Figure 5 describes the DSC curves of as-prepared and crosslinked FK/PVA/PEO nanofiber membranes. The endothermic peaks of the FK/PVA/PEO nanofiber membranes were observed between 50 and 80 °C. The peaks at ~60 °C and ~80 °C correspond to the melting of the PEO crystalline phase and the evaporation of water, respectively [26,27]. The peaks at ~200 °C and ~270 °C refer to the PVA melting and FK endothermic peaks, respectively [26]. Both PVA melting and FK endothermic peaks have been shifted towards higher temperature after vapor-assisted crosslinking modification of FK/PVA/PEO nanofiber membranes. The results indicate that the thermal stability of crosslinked FK/PVA/PEO nanofiber membrane has been improved, which is consistent with the TGA results, given in Figure 4.

### 3.4. Hydrophobicity Analysis

**Figure 6:**
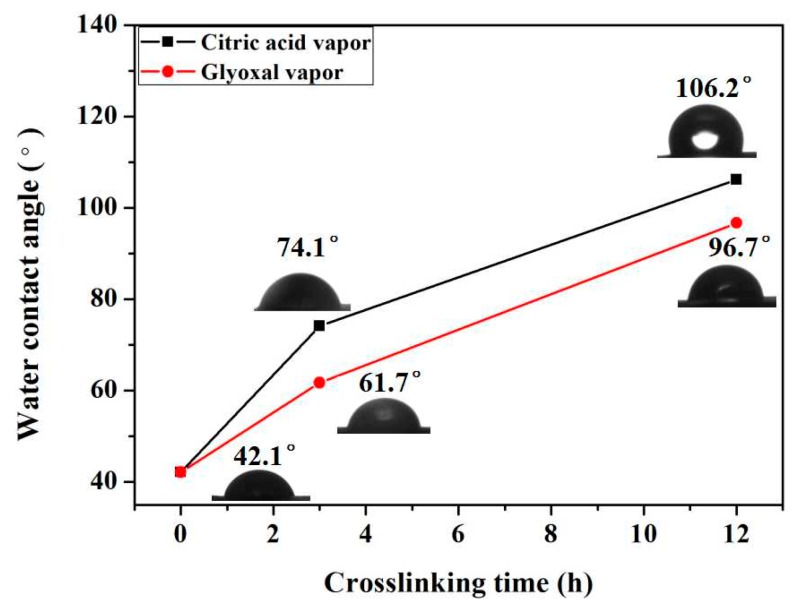
The water contact angle of as-prepared and crosslinked FK/PVA/PEO nanofiber membranes.

Water contact angle (WCA) is an important measurement to assess the water absorption and swelling properties of polymeric membranes. The WCA of the as-prepared and crosslinked FK/PVA/PEO nanofiber membranes is shown in Figure 6. The WCA of as-prepared FK/PVA/PEO nanofiber membrane is 42.1°, whereas the WCA dramatically increased to 106.2° and 96.7° after vapor-assisted crosslinking modification for 12 h with citric acid and glyoxal, respectively. Moreover, the WCA has shown a direct relationship with reaction time for both crosslinkers. The results suggest that the crosslinking has significantly improved the water resistance of the nanofiber membrane, which can be attributed to the chemical interactions of membrane materials and crosslinkers. In addition, the water resistance of the citric-treated membranes is higher than glyoxal-treated membranes.

### 3.5. Mechanical Characterization

**Figure 7:**
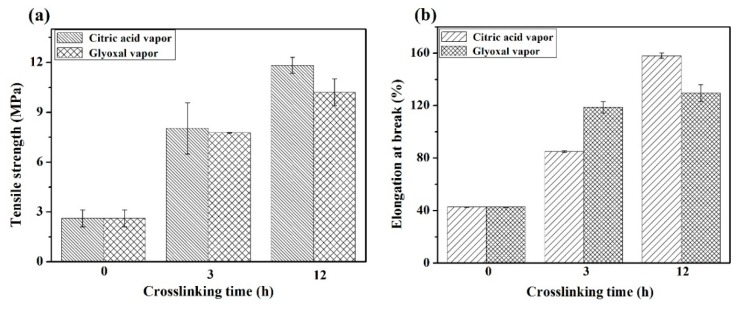
The mechanical properties of as-prepared and crosslinked FK/PVA/PEO nanofiber membranes: (**a**) tensile strength (σ_b_); and (**b**) elongation at breakage point (ε_b_).

The mechanical properties of as-prepared and crosslinked FK/PVA/PEO nanofiber membranes are presented in Figure 7. The mechanical properties, such as tensile strength (σ_b_) and elongation at breakage point (ε_b_), were deduced from linear stress-strain curves. Figure 7 shows that the σ_b_ and ε_b_ of the as-prepared FK/PVA/PEO nanofiber membrane are 2.62 MPa and 42.75%, respectively. Both σ_b_ and ε_b_ have increased after crosslinking and showed a direct relationship with crosslinking time. The σ_b_ of the nanofiber membranes, treated with citric acid vapors and glyoxal vapors for 12 h, are about 4.5 times and 3.8 times higher than that of the as-prepared FK/PVA/PEO nanofiber membrane, respectively. Furthermore, the ε_b_ of 12h-C and 12h-G is about 3.7 times and 3.0 times higher than that of the as-prepared FK/PVA/PEO nanofiber membrane, respectively. The results indicate that the σ_b_ and ε_b_ of nanofiber membrane significantly increased by vapor crosslinking modification, which may be caused due to the reactions between the crosslinkers and the FK/PVA/PEO nanofiber membrane. As a result of this interaction, a large number of crosslinking chains were formed and enhanced mechanical properties were observed [28]. Moreover, Figure 7 reveals that the mechanical properties of citric-treated membranes are better than glyoxal-treated membranes, which may be ascribed to the larger diameter of the citric-treated nanofibers. Additionally, the error bars in Figure 7 were achieved by measuring approximately 20 pieces of independently-prepared nanofiber membranes. As can be seen from Figure 7 shown through error bars, the reproducibility of mechanical characterization is good, which implies the preparing procedure is good and reliable. These results prove that the mechanical properties of crosslinked FK/PVA/PEO nanofiber membranes have been effectively improved by vapor-assisted crosslinking processing.

## 4. Conclusions

Herein, we have demonstrated the successful synthesis of an FK/PVA/PEO three-component nanofiber membrane by electrospinning and a novel vapor-assisted crosslinking process. The feather-derived-keratin (FK), PVA, and PEO were blended and electrospun, following by crosslinking with citric acid and glyoxal vapors. The results show that the average diameter of the nanofibers increased from 223 ± 36 nm to 342 ± 58 nm and 304 ± 55 nm for the as-prepared, citric-treated, and glyoxal-treated membranes, respectively. Similarly, the crosslinked membranes have shown significantly improved thermal stability and water resistance. The tensile strength (σ_b_) of citric-treated and glyoxal-treated membranes was about 4.5 times and 3.8 times higher than that of as-prepared FK/PVA/PEO nanofiber membrane, respectively. Furthermore, the elongation at breakage point (ε_b_) is about 3.7 times and 3.0 times higher than that of as-prepared FK/PVA/PEO nanofiber membrane. The results exhibit excellent compatibility of individual components, thermal stability, water resistance and mechanical properties of crosslinked FK/PVA/PEO nanofiber membranes. Moreover, the performance of citric-treated membranes was better than the glyoxal-treated membranes. This study reveals that a non-toxic crosslinking agent, citric acid, can be used for effective crosslinking of keratin-based membranes, which is promising for the successful realization of these materials in a wide range of applications.

## Figures and Tables

**Table 1:** The average diameter of the FK/PVA/PEO nanofiber membranes with different vapor-assisted crosslinking modifications.

FK/PVA/PEO Membrane	Average Diameter (nm)
without treatment	223 ± 36
3h-C	255 ± 52
12h-C	342 ± 58
3h-G	291 ± 45
12h-G	304 ± 55

**Table 2:** Chemicals and materials.

Name Grade/Purity	Supplier
Chicken feathers	Waste Market
Peracetic acid Analytical reagent (AR)	Tianjin Fuchen Chemical Reagents Factory
PVA Degree of alcoholysis: 87–89%	Shanghai Aladdin Biochemical Polytron Technologies Inc.
PEO Molecular weight: 400,000 Da	Shanghai Aladdin Biochemical Polytron Technologies Inc.
Citric acid Analytical reagent (AR)	Tianjin Fuchen Chemical Reagents Factory
Glyoxal solution 40 wt %	Shanghai Aladdin Biochemical Polytron Technologies Inc.
